# Modulation of Gut Microbiota Profile and Short-Chain Fatty Acids of Rats Fed with Taro Flour or Taro Starch

**DOI:** 10.1155/2020/8893283

**Published:** 2020-08-18

**Authors:** Ingrid S. Surono, Koen Venema

**Affiliations:** ^1^Food Technology Department, Faculty of Engineering, Bina Nusantara University, Jakarta 11480, Indonesia; ^2^Centre for Healthy Eating & Food Innovation, Maastricht University–Campus Venlo, St. Jansweg 20, 5928 RC, Venlo, Netherlands

## Abstract

To investigate the effect of flour and starch of the Indonesian native tuber “taro” on the composition and activity of the gut microbiota in diabetic rats, streptozotocin (STZ)-induced diabetic rats were fed normal chow (AIN), or AIN in which corn starch was replaced by either taro flour or purified taro starch for 4 weeks. Fecal samples were collected at baseline and after 4 weeks, and the composition of microbial communities was measured using 16S rRNA sequencing, while SCFAs were measured using ion chromatography. Bodyweight declined upon DM induction with STZ. Feeding taro starch led to a lower reduction in bodyweight than feeding taro starch, but this was only significant for taro starch in weeks 2, 3, and 4 (*p* = 0.02, *p* = 0.01, and *p* < 0.01, respectively). Both taro starch and taro flour induced changes in the gut microbiota composition compared to AIN, which were different for taro flour and taro starch. *Bifidobacterium*, *Sutterella*, and *Prevotella* were markers for taro flour feeding, while *Anaerostipes* was a marker for taro starch feeding. Induction of diabetes also led to changes in the microbiota composition. Random Forest correctly predicted for 16 of 18 samples whether rats were diabetic or not and correctly predicted 6 of 12 microbiota samples belonging to either taro flour- or taro starch-fed groups, indicating also some significant overlap in the substrate, as expected. Taro starch and taro flour both led to a significant increase in the fecal concentrations of acetate, propionate, and butyrate.

## 1. Introduction

Cocoyam or taro belongs to the monocotyledonous family Araceae (the aroids) and is an important root crop and is therefore cultivated and used for food in various parts of the humid tropics and subtropics [1]. It adapts well to different agroclimatic conditions [2] and is cultivated and traditionally used as a food crop by several ethnic communities in Borneo [3]. Kreike et al. [4] reported that Indonesia has the highest taro diversity in the world, and apart from Borneo, it is found in areas of Java, Sumatra, and Sulawesi [5]. The utilization of taro is also related to the culture of a region; hence, taro is very important for community life [6], and it is utilized as staple foods or as a snack, processed with traditional recipes based on the local culture of ethnic groups. Taro is also one of the staple foods of Papua communities in the highland due to the simple cultivation technique [7].

The aroid root crop species belong to two subfamilies: Colocasioideae (*Alocasia macrorrhiza*, *Colocasia esculenta*, and *Xanthosoma sagittifolium*) and Lasioideae (*Amorphophallus campanulatus* and *Cyrtosperma chamissonis*) and play an important role as staple foods in Asian culture [8]. *Colocasia esculenta* (L.) Schott and *Xanthosoma sagittifolium* (L.) Schott are the two most important genera that are generally grown for food. *Xanthosoma sagittifolium* was found to have low glycemic index (GI), a classification of food based on the blood glucose response to a food relative to a standard glucose solution [9]. Low glycemic foods control the release of glucose into the bloodstream at a more steady and sustained rate, without glucose peaks, keeping the body's metabolic processes and energy levels balanced. Hence, it would benefit those who are already suffering from hyperglycemia since these would help in the proper control and management of blood sugar.

Taro flour and starch are potential sources to substitute for wheat flour, which has been widely used in Indonesia, but depends on imports. The use of taro flour and starch leads to support the efforts to diversify (ethnic) foods as well as food security in Indonesia.

Type 2 diabetes mellitus (T2DM) needs dietary considerations due to the vital role of food in the prevention and treatment to influence postprandial (hyper)glycaemia in T2DM. The global incidence of T2DM is predicted to reach 360 million cases by the year 2030 [10], and therefore, there is an urgent need for investigations into the antihyperglycaemic effects of plant foods.

The importance of the intestinal microbiota in host health and pathogenesis of several noncommunicable diseases such as cancer, obesity, and T2DM, and even mental disorders is recognized [11, 12]. Cross-sectional case-control studies have revealed microbial dysbiosis in T2DM patients [13, 14] indicating a possible contribution of the microbiota to disease development.

The growing interest of public health concerning the treatment and prevention of diseases such as T2DM has led to functional foods being explored [15], and it has been shown that functional foods contribute to the improvement of overall health and reduce the occurrence of diseases [16].

The aims of the present study were to investigate the effect of the ethnic taro flour and taro starch on the modulation of the gut microbiota, by 16S rRNA gene profiling and measuring short-chain fatty acid (SCFA) concentrations in rats.

## 2. Materials and Methods

### 2.1. Materials

Streptozotocin (STZ) was purchased from Enzo Life Sciences (Farmingdale, NY, USA). 10% ketamine and 2% xylazine (used for anesthesia) were from Kepro (Deventer, the Netherlands) and Interchemie (Castenray, the Netherlands), respectively. Taro starch “HASIL BUMIKU” was produced by Kusuka Ubiku, Bantul, Yogyakarta, Central Java, Indonesia, and taro flour “NAYA” was produced by Primanaya, Bogor, West Java, Indonesia. L-cystine was from Now Foods (Bloomingdale, IL, USA), choline bitartrate was from Nature's Way (Miami, FL, USA), and mineral mix and vitamin mix (as multivitamin Fitkom) were purchased from a pet shop in Bogor.

Purified Rodent Diet AIN-93M, a modified AIN-76A standard diet (American Institute of Nutrition) [17], was used as a control diet. This diet was modified with test products in the form of taro flour and native taro starch, by replacing corn starch with the taro products (Table 1).

### 2.2. Measurement of Resistant Starch

Resistant starch was determined using the Megazyme resistant starch assay kit (K-RSTAR; Megazyme, Irishtown, Ireland) according to the specifications of the manufacturer.

### 2.3. Animals and Housing

Male Sprague Dawley rats (6–8 weeks, weighing approximately 150–200 g at the beginning of the experiment) were purchased from the experimental animal laboratory of the National Agency of Drug and Food Control (Jakarta, Indonesia). They were kept in rooms with controlled temperature (23 ± 3°C), relative humidity of 65–75%, and a 12 h/12 h light/dark cycle (lights on from 7:00 AM to 7:00 PM). The bedding of the cages consisted of wood shavings that were changed every 1–3 days throughout the experiment and prior to the induction of experimental diabetes for those in the diabetic group (see below). The rats were housed in individual plexiglass cages measuring 37.1 × 23.8 × 21.6 cm and allowed to adapt for 14 days, and during the acclimatization period, all rodents were given ad libitum access to water and commercially available rat normal pellet diet (NPD) purchased from a local market, prior to the dietary manipulation. This study was approved by the Bimana Indomedical Animal Care and Use Committee and conformed to the Guidelines for Care and Use of Laboratory Animals of the Bimana Indomedical, Bogor, Indonesia. Efforts were made to minimize the number of animals used as well as their suffering.

### 2.4. Treatment with Streptozotocin (STZ)

Nine rats were not injected with STZ and functioned as normal (nondiabetic) rats. For STZ treatment, in fifteen rats, diabetes was induced by a single intraperitoneal injection of 55 mg/kg STZ solutions, prepared immediately before use, in 0.1 M citrate buffer (pH 4.5). The second dose was administered on day 14 following the first STZ dose, at 60 mg/kg doses. The goal was to generate a prediabetic hyperglycemic state [18, 19]. At days 2 and 9 after the STZ injection, fasting and postprandial blood glucose levels were measured using a FreeStyle Easy Touch glucose meter (Abbott Diabetes Care Inc., Alameda, CA, USA) from blood taken from the tip of the tail. The rats with a fasting glucose of 100 mg·dl^−1^ and/or postprandial blood glucose levels of ≥140 mg dl^−1^ were considered as type 2 diabetic. Of this group of 15 rats, 9 eight-week-old rats of 190–220 g with established T2DM (DM hereon after) were selected for the intervention study with taro flour and taro starch.

The rats consumed Purified Rodent Diet AIN-93M, a modified AIN-76A standard diet (American Institute of Nutrition) [17] as a control and mineral water from water bottles ad libitum.

### 2.5. Feeding Treatment Groups

The rats in the two groups (diabetic (DM) rats and nondiabetic (non-DM) rats (*n* = 9 each)) were divided into three treatment groups (*n* = 3 each): AIN-93M (hereon after AIN), modified AIN by replacing corn starch with taro flour, and modified AIN by replacing corn starch with taro starch. Sucrose and cellulose were replaced with maltodextrin (Table 1). Feeding adaptation was conducted for 4 days, with 25, 50, 75, and 100% dietary intervention formulation, respectively, prior to four-week dietary treatments. Bodyweight was measured weekly. In addition, fasting blood samples were taken for measurements of fasting glucose (FreeStyle Easy Touch glucose meter; Abbott Diabetes Care Inc.). Moreover, blood samples were taken weekly from the tip of the tail and stored at −20°C for measurements of GLP-1 and PYY. Also, freshly voided fecal pellets were collected weekly and stored at −80°C for the determination of gut microbiota composition.

### 2.6. Measurement of GLP-1 and PYY

GLP-1 and PYY were measured using ELISA, using kits from Crystal Chem (Elk Grove Village, IL, USA) according to the instruction provided by the manufacturer. Detection limits were 1.24 pM and 0.15 ng/mL for GLP-1 and PYY, respectively.

### 2.7. Fecal DNA Extraction and Microbial 16S rRNA Gene Amplicon Sequencing

Genomic DNA extraction was performed using the Quick-DNA™ Fecal/Soil Microbe Miniprep Kit (Zymo Research) according to the manufacturer's instructions. Illumina 16S rRNA gene amplicon libraries were generated and sequenced at BaseClear (Leiden, the Netherlands). In short, barcoded amplicons from the V3-V4 region of 16S rRNA genes were generated using a 2-step PCR. 10–25 ng genomic DNA was used as a template for the first PCR with a total volume of 50 *μ*l using the 341F (5′-CCTACGGGNGGCWGCAG-3′) and the 785R (5′-GACTACHVGGGTATCTAATCC-3′) primers appended with Illumina adaptor sequences. PCR products were purified (QIAquick PCR Purification Kit), and the size of the PCR products was checked on a Fragment Analyzer (Advanced Analytical, Ankeny, USA) and quantified by fluorometric analysis. Purified PCR products were used for the second PCR in combination with sample-specific barcoded primers (Nextera XT index kit, Illumina). Subsequently, PCR products were purified, checked on a Fragment Analyzer, and quantified, followed by multiplexing, clustering, and sequencing on an Illumina MiSeq with the paired-end (2x) 300 bp protocol and indexing. The sequencing run was analyzed with the Illumina CASAVA pipeline (v1.8.3) with demultiplexing based on sample-specific barcodes.

The sequencing was carried out using the Illumina MiSeq system, and later, the sequences were converted into FASTQ files using BCL2FASTQ pipeline version 1.8.3. The quality cut was applied based on the quality level of Phred (Phred quality score). Quantitative Insights Into Microbial Ecology (QIIME) software package (1.9.0) was used for microbial analyses [20]. The sequences were classified using Greengenes (version 13.8) as a reference 16S rRNA gene database. Linear discriminant analysis effect size (LEfSe) [21] was used to find biomarkers between groups using relative abundances from the operational taxonomic unit (OTU) tables generated in QIIME.

### 2.8. Statistical Analysis

The software package R (3.5.0) (R Core Team, 2013) was used to determine correlations between OTUs and variables in the study. These statistical analyses were performed with RStudio. Spearman correlations were calculated between the relative abundance of OTUs and continuous variables (e.g., SCFA concentrations). Kruskal–Wallis correlation was determined between the relative abundance of OTUs and noncontinuous values (e.g., diet). Multiple comparison was corrected using the false discovery rate (FDR). To evaluate differences between two groups, Student's *t*-tests were used. *p* values or *q*-values (adjusted *p* values) were considered significantly different at <0.05.

## 3. Results

### 3.1. Bodyweight, Fasting Glucose, GLP-1, and PYY

**Figure 1:**
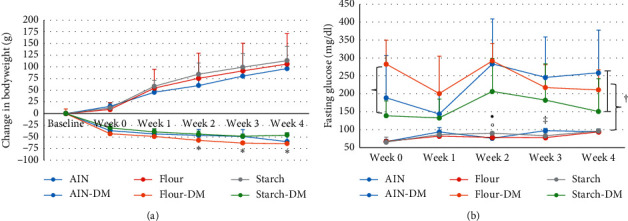
(a) Changes in bodyweight development of the non-DM and DM rats over the 4-week period. (b) Fasting glucose of the non-DM and DM rats over the 4-week period.

Changes in bodyweight are shown in Figure 1(a). As expected, bodyweight declined upon DM induction with STZ. Feeding taro starch led to a lower reduction in bodyweight than feeding taro flour, but this was only significant for taro starch in weeks 2, 3, and 4 (*p* = 0.02, *p* = 0.01, and *p* < 0.01, respectively). There was no significant difference between AIN and the taro-based diets. Both taro flour and taro starch led to a higher increase in bodyweight of the non-DM rats, with taro starch leading to the highest increase, but due to large variation within the groups, this did not reach significance. Feed intake was the same for DM rats, so this could not explain the difference in bodyweight change. Feed intake for the non-DM rats showed that AIN intake was significantly lower compared to taro flour intake at weeks 1, 2, and 3 (all *p* < 0.05) (average over the 3 weeks 6.7 ± 1.4 g/day vs. 9.5 ± 0.8 g/day, respectively), but there was no significant difference with taro starch (9.0 ± 2.0 g/day).

As expected, fasting blood glucose (Figure 1(b)) was significantly higher for DM rats (*p*=3*∗*10^−12^), but due to large variation within the groups, this was only significantly different between taro flour and taro starch at baseline. For the non-DM rats, differences were small, but significant at week 2 between AIN and taro starch (*p*=0.03) and between taro flour and taro starch (*p*=0.003) and at week 3 between AIN and taro flour (*p*=0.011).

GLP-1 concentrations in blood were similar for the non-DM and DM groups at baseline (3.4–3.5 pM). For the non-DM rats that were fed AIN, this stayed the same throughout the 4-week period, but the GLP-1 concentrations increased for taro flour-fed and taro starch-fed rats after the second week almost linearly to 35.4 (*p*=0.013) and 20.2 pM (*p* ≤ 0.01), respectively. This was not observed for the DM rats. The GLP-1 concentrations for the AIN-fed DM rats were relatively stable over time and were periodically higher for the taro-fed rats, but not consistently (data not shown).

PYY concentrations were higher at baseline for the non-DM rats (∼3.5 ng/mL vs. 0.7 ng/mL for DM rats), but dropped to ∼1.5 ng/mL after 4 weeks of intervention irrespective of the diet. For the DM rats, the concentrations were lower after STZ-induced DM (∼0.7 ng/mL) but increased after the first week to ∼1.5 ng/mL and remained relatively stable after that. After 4 weeks, there was a small but significant difference between AIN and taro flour (1.66 ± 0.20 vs. 1.16 ± 0.12, *p*=0.036) and a trend for a difference between AIN and taro starch (1. .66 ± 0.20 vs. 1.54 ± 0.16, *p*=0.056), although at earlier weeks, the concentrations for taro-fed rats were (nonsignificantly, due to large variation between animals) higher (data not shown).

### 3.2. Resistant Starch Content of the Taro Flour and Taro Starch

The resistant starch content of the flour was 3.2%. Due to the isolation process, the resistant starch content of the purified starch increased to 32.8%. This meant that much more resistant starch arrived in the colon of the taro starch-fed rats, where it was a substrate for the gut microbiota.

### 3.3. Fecal Microbial Composition of the Rats after the Intervention

**Figure 2:**
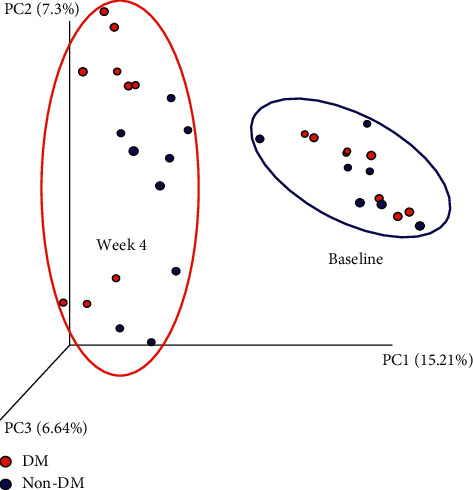
*β*-diversity analysis (unweighted UniFrac) of baseline and week 4 microbiota of DM and control (non-DM) rats. Although the rats at baseline are labelled DM, they have not been induced with STZ yet, but would become DM later in the study.

The V3-V4 region of the 16S rRNA gene was amplified and sequenced to determine the compositional changes of the microbiota upon intervention with AIN, taro starch, or taro flour. The baseline microbiota was taken to be the one after T2DM induction by STZ in the diabetic rats and the same moment after sham induction in the nondiabetic rats. At this time point, i.e., at baseline before the intervention with the 3 diets, the microbiota of the rats was similar (Figure 2; baseline), despite induction of DM by STZ in half of the animals.

**Figure 3:**
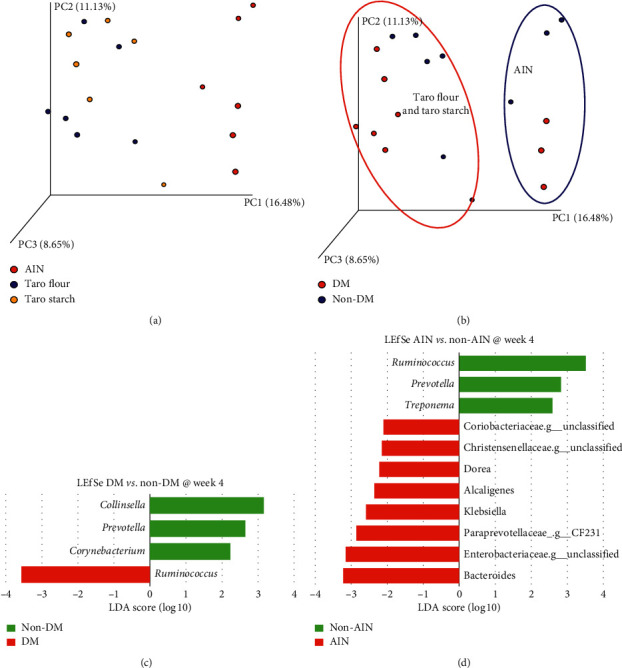
(a, b) *β*-diversity analysis (unweighted UniFrac) of week 4 microbiota of DM and control (non-DM) rats on the different diets. (a) Labelling of the samples according to diet; (b) labelling of the samples according to DM status. (c, d) Linear discriminant analysis effect size (LEfSe) of AIN- vs. taro-based feeding (c) and DM status (d) at week 4.

After 4 weeks of intervention with the 3 diets, the microbiota was clearly different (*β*-diversity) than at baseline (Figure 2; week 4), irrespective of whether rats were DM or non-DM. Further comparisons were made between the different groups at this time points (4 weeks after dietary intervention). From Figure 3(a), it is clear that treatment with either taro flour or taro starch induced a different microbiota than when rats were fed AIN-93M. Moreover, Figure 3(b) shows that the presence of DM segregates the rats into two groups within the AIN-93M-fed rats and into two groups within the taro-fed rats.

**Figure 4:**
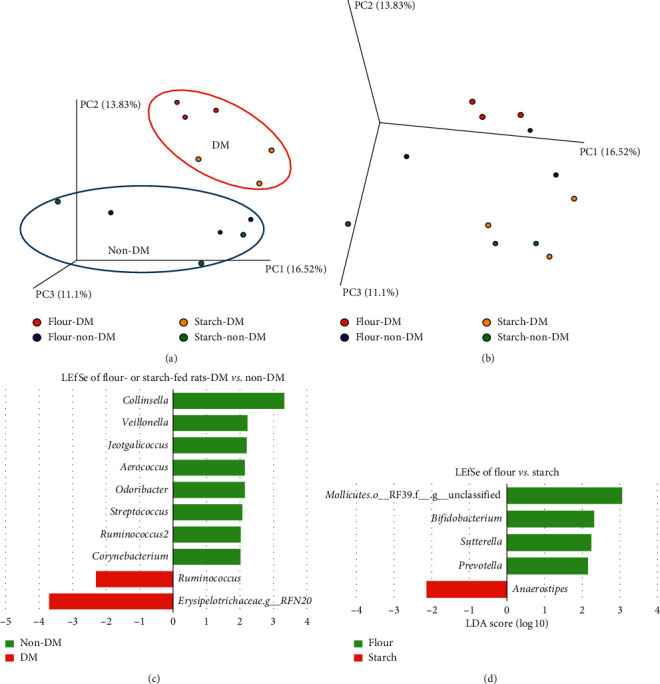
(a, b) *β*-diversity analysis (unweighted UniFrac) of week 4 microbiota of DM and control (non-DM) rats on the two taro-based diets. (a) Representation to show separation between DM and non-DM; (b) same graph as in (a), but rotated to show separation between taro starch and taro flour. (c, d) Linear discriminant analysis effect size (LEfSe) of DM status (c) and taro starch- vs. taro flour-fed rats at week 4 (d) in taro-fed rats (excl. AIN).

This DM-induced segregation is also observed within either the taro flour- or the taro starch-fed group (Figure 4(a)). Moreover, Figure 4(b) shows that taro starch feeding led to a different microbiota compared to taro flour feeding. As the research aimed to study the microbiota changes after taro flour or taro starch intervention, we focused on this, in conjunction with T2DM.

**Figure 5:**
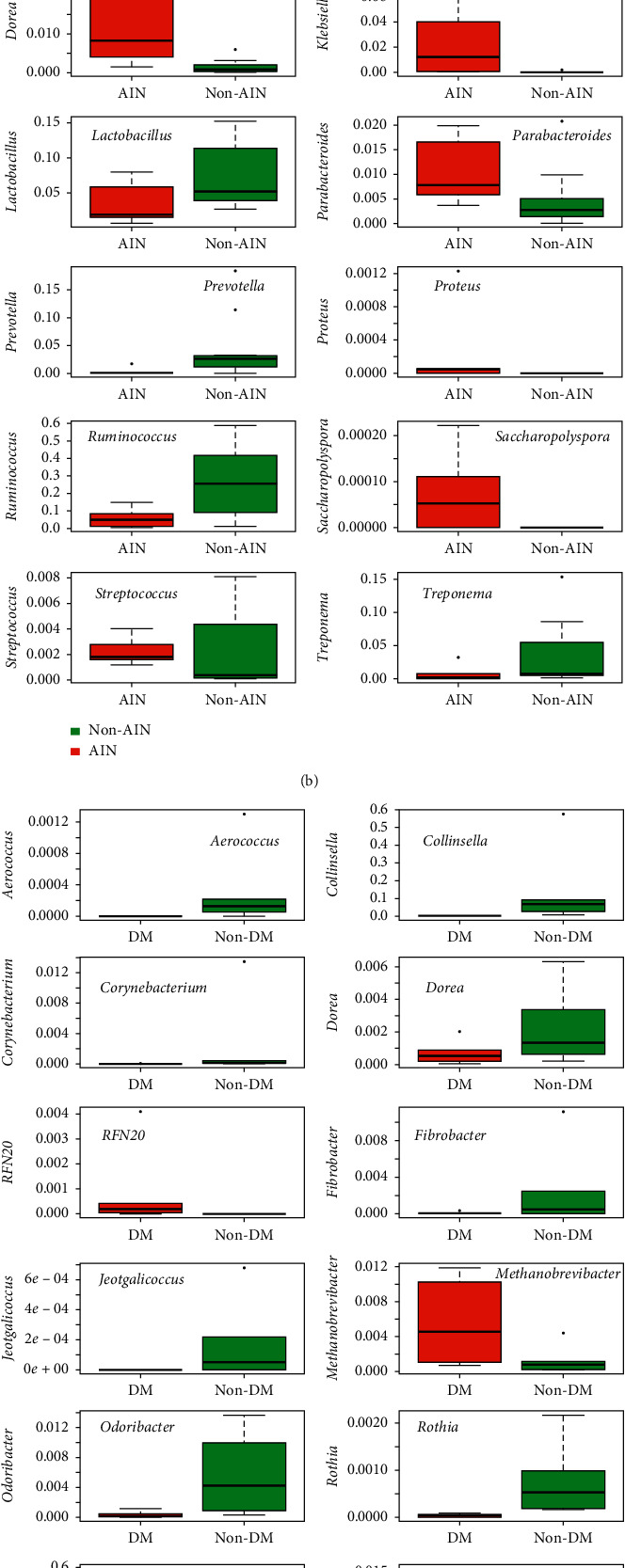
Box plots of those OTUs that were different (using LEfSe, Random Forest (top 10 OTUs) and Kruskal–Wallis correlation) between the microbiota from DM and non-DM rats at week 4 (a), AIN- and non-AIN-fed rats at week 4 (b), between the microbiota from DM and non-DM rats from only the taro flour- and taro starch-fed rats (c), and between the microbiota from the taro flour- or taro starch-fed rats (d).

Linear discriminant analysis effect size (LEfSe) [21] was used to find OTUs at the genus level that were different between groups at week 4. On top of that, Kruskal–Wallis correlation analyses were performed in R.  Figure 3(c) shows the LEfSe results at the genus level between the DM and non-DM rats at week 4. *Ruminococcus* was a marker for the microbiota of DM rats, while *Collinsella*, *Prevotella*, and *Corynebacterium* were markers for the microbiota of non-DM rats (see Figure 5 for box plots).

**Figure 6:**
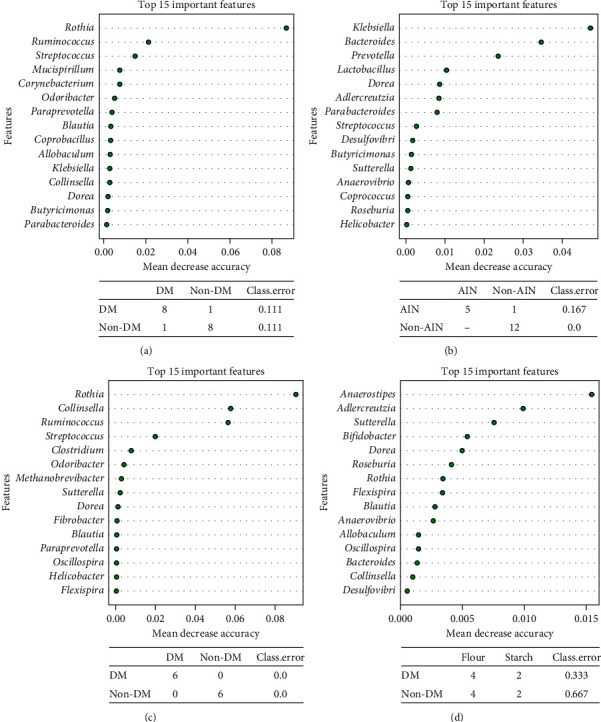
Random Forest prediction (run in METAGENassist) of samples in week 4 belonging to either DM or non-DM rats (a) and AIN- or taro-fed (non-AIN) rats (b). (c) Prediction of DM and non-DM of taro-fed rats only. (d) Prediction of starch-fed or flour-fed rats. The top 15 markers are shown. Of these, the top 10 have been plotted in Figure 5 (together with markers identified by LEfSe and Kruskal–Wallis correlation).

Kruskal–Wallis correlation indicated differences in *Rothia*, *Staphylococcus*, and *Aerococcus* (all higher in non-DM; all *q*-values: 0.025). Using Random Forest (RF) algorithms, it was possible to correctly predict for 16 out of 18 samples whether a sample would be classified as DM or non-DM (Figure 6(a)). The top 3 OTUs that contributed most to the RF prediction were *Ruminococcus*, *Rothia*, and *Streptococcus* (higher in non-DM; Figure 6(a)). Similarly, comparing rats that were fed AIN- or taro-based products (flour or starch), LEfSe indicated *Ruminococcus*, *Prevotella*, and *Treponema* to be more abundant in taro-fed rats (non-AIN), while an unclassified genus of the Coriobacteriaceae family, an unclassified genus of the Christensenellaceae family, *Dorea*, *Alcaligenes*, *Klebsiella*, and *CF231* of the family Paraprevotellaceae, an unclassified genus of the family Enterobacteriaceae, and *Bacteroides* were more abundant in AIN-fed rats at week 4 (Figure 3(d)). Kruskal–Wallis correlation analyses overlapped partly with the genera *CF231*, *Bacteroides*, *Klebsiella*, the unclassified genus of the Christensenellaceae family, and the unclassified genus of the family Enterobacteriaceae (all higher in AIN, all *q*-values: 0.045), and in addition, the genera *Saccharopolyspora* (higher in AIN; *q*-value is 0.045) and *Proteus* (higher in non-AIN; *q*-value: 0.045; Figure 5(b)). RF correctly predicted for 17 out of 18 samples whether the rats were fed AIN or one of the taro-based diets (Figure 6(b)). The top 3 OTUs that contributed most to the RF prediction were *Klebsiella*, *Bacteroides* (both higher in AIN), and *Prevotella* (higher in non-AIN; Figure 6(b)).

Focusing on the rats that received the taro-based treatments (either flour or starch), the differences observed in *β*-diversity were also investigated. LEfSe indicated a number of OTUs at the genus level that were different between DM and non-DM rats (Figure 4(c)). The genera *Collinsella*, *Veillonella*, *Jeotgalicoccus*, *Aerococcus*, *Odoribacter*, *Streptococcus*, *Ruminococcus* (within the Lachnospiraceae family), and *Corynebacterium* were markers for non-DM rats, while *Ruminococcus* (within the Ruminococcaceae family) and the genus *RFN20* of the Erysipelotrichaceae were markers for DM rats. Kruskal–Wallis analysis did not reveal any correlations with a *q*-value of <0.05. We used this strict cutoff to only focus on differences that are expected to be real after multiple comparisons. RF correctly predicted 12 out of 12 samples. The top 3 OTUs were *Rothia*, *Collinsella*, and *Ruminococcus* (Figure 6(c)). Two of these were also amongst the top 3 for the samples including AIN. The differences in microbiota modulation between the two taro-based substrates used are indicated by LEfSe in Figure 4(d). Together with an unclassified genus of the RF39 order of the Mollicutes class, the genera *Bifidobacterium*, *Sutterella*, and *Prevotella* were markers for taro flour feeding, while *Anaerostipes* was a marker for taro starch feeding. *Anaerostipes* and *Sutterella* were among the top 3 OTUs of RF, with *Bifidobacterium* on the 4th place. However, RF only predicted 50% of the samples correctly (Figure 6(d)). Also here, Kruskal–Wallis analysis did not reveal any correlations with a *q*-value of <0.05.

**Figure 7:**
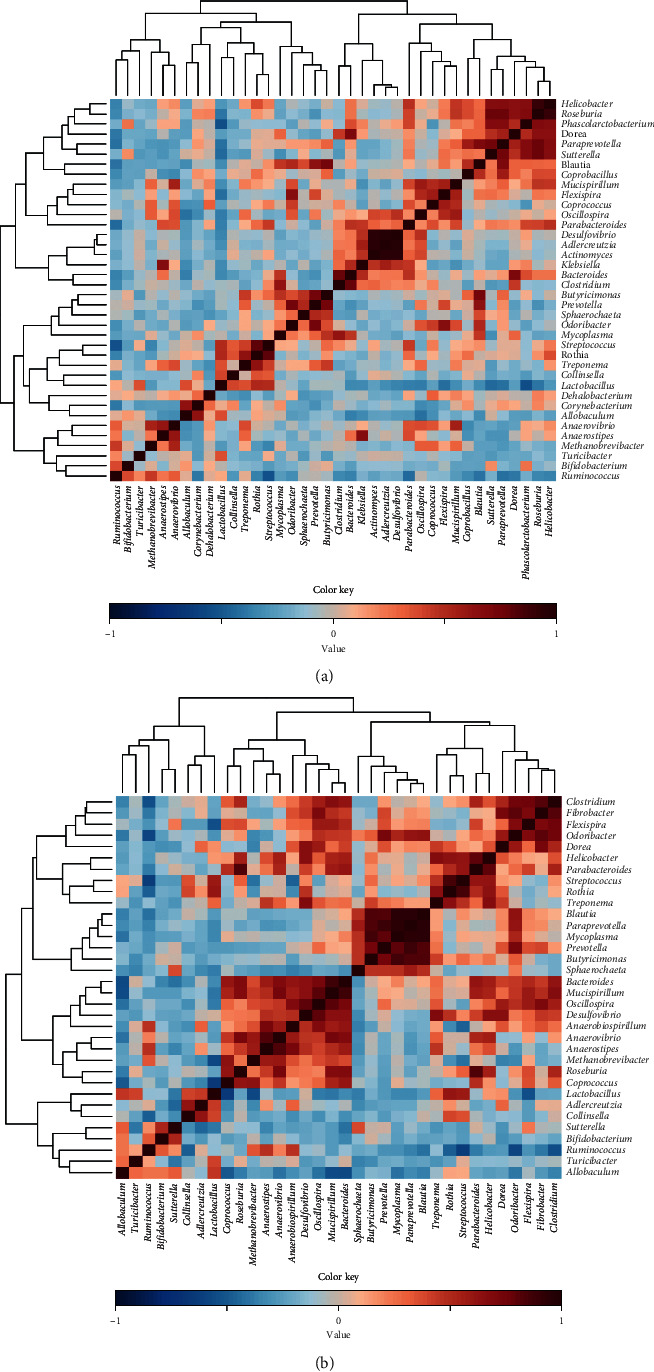
Correlation analysis (run in METAGENassist) of the microorganisms present in all samples at week 4 (a) or in the week 4 samples of the taro starch- and taro flour-fed rats (b).

The co-occurrence of OTUs in the dataset of week 4 (Figure 7(a)) or the 4-week dataset of the taro-fed animals (excluding AIN) (Figure 7(b)) shows that due to a different microbiota composition upon AIN feeding (Figures 3(a) and 3(d)), there are quite some differences in co-occurrences between the datasets.

### 3.4. SCFA Concentrations

Overall, SCFA concentrations in fecal pellets did not differ between baseline and week 4 (*p* > 0.14). Also, when considering all rats together (baseline + week 4), there was no difference in SCFA between DM and non-DM rats, or between AIN- and non-AIN-fed rats.

Focusing on week 4, all three SCFAs were significantly different when comparing AIN- to taro-fed rats, AIN- to taro flour-fed rats, and AIN- to taro starch-fed rats. In all cases, SCFAs were significantly higher in the taro-fed rats compared to AIN (*p* < 0.01 for acetate, propionate, and butyrate). There was no significant difference between taro flour- and taro starch-fed rats (Table 2). Spearman correlation did not reveal any correlation between OTUs and SCFA in the full dataset, but showed a negative correlation for both propionate and butyrate with an uncharacterized Enterococcaceae genus at week 4 (*q*-value: 0.003, rho = −0.83 for propionate; *q*-value: 0.02, rho = −0.78 for butyrate).

## 4. Discussion

LEfSe indicated *Ruminococcus*, a butyrate producer, to be a marker of DM in the gut microbiota of the rats. On the other hand, *Collinsella*, *Corynebacterium*, and *Prevotella* were found to be markers for the gut microbiota of non-DM rats. The genus *Collinsella* is often described as a pathobiont-producing lactate (instead of butyrate or other SCFAs) [22]. Lactate can be converted into propionate or butyrate through cross-feeding by other members of the gut microbiota [23]. In humans, the abundance of *Collinsella* is increased in T2D patients when compared to healthy control subjects [24]. Likewise, *Corynebacterium* also produces lactic acid instead of SCFA, while *Prevotella* produces propionic acid. Despite the differentiation of these microbial markers for (non-)DM, SCFA concentrations in fecal pellets of DM rats were not significantly different from non-DM rats.

Principal coordinate analysis (PCoA) revealed a significant microbiome separation into two distinct clusters after 4 weeks of intervention between taro- and AIN-fed rats (Figure 3(b)). LEfSe indicated that in taro-fed rats, *Ruminococcus*, *Prevotella*, and *Treponema*, producing, respectively, butyrate, propionate, and acetate, dominated. In AIN-fed rats, an unclassified genus of the Coriobacteriaceae family, an unclassified genus of the Christensenellaceae family, *Dorea*, *Alcaligenes*,*Klebsiella*, and *CF231* of the family Paraprevotellaceae, an unclassified genus of the family Enterobacteriaceae, and *Bacteroides*, mostly Gram-negative bacteria, were more abundant. There was a significantly higher concentration of fecal SCFA (butyrate, propionate, and acetate) in taro-fed rats than in AIN-fed rats (Table 2). The starch content (60.5%) and high dietary fiber content (15.4% insoluble and 2.8% soluble fiber) in *Xanthosoma sagittifolium* [25] could contribute in supporting the growth of the observed OTUs and the accompanying increased SCFA production.

Comparing the two taro-fed groups (starch vs. flour), an unclassified genus of the RF39 order of the Mollicutes class and the genera *Bifidobacterium*, *Sutterella*, and *Prevotella* were markers for taro flour feeding, while *Anaerostipes* was a marker for taro starch feeding. *Anaerostipes* and *Sutterella* were also among the top 3 OTUs of random forest prediction, with *Bifidobacterium* on the 4th place. Despite these differences in the abundance of these microbes, no significant changes in SCFA production were observed, although there was a trend for higher acetate in the flour-fed rats (*p* = 0.07).

Short-chain fatty acids (SCFAs) play an important role in gut physiology. Although fecal SCFA concentrations do not necessarily mean higher SCFA production (it could also be lower absorption by the gut epithelium, leading to higher excretion), it is well known that starch leads to higher SCFA production, particularly butyrate [26–28]. SCFAs have been shown to be beneficial in obesity and (pre)diabetes. For instance, infusion of acetate or SCFA mixtures in the distal colon has led to increased energy expenditure and fat oxidation [29, 30].

## 5. Conclusion

In conclusion, taro feeding, in the form of either flour or starch, leads to a change in gut microbiota composition, accompanied with an increase in SCFA production (compared to AIN). As such, products containing the ethnic taro tuber *Xanthosoma sagittifolium* may be a good alternative for wheat-based food products in Indonesia. SCFAs have been shown to beneficially affect obesity and (pre)diabetes and may help in preventing a further increase in this endemic global problem.

## Figures and Tables

**Table 1:** Composition of different formulations.

Composition	AIN-93M (% by weight)	Taro flour (% by weight)	Native taro starch (% by weight)
Corn starch	59.27	—	—
Taro flour	—	59.27	—
Native taro starch	—	—	59.27
Casein	14.00	14.00	14.00
L-Cystine	0.18	0.18	0.18
Maltodextrin	15.50	24.00	24.00
Sucrose	5.00	—	—
Cellulose	3.50	—	—
Soybean oil	0.18	0.18	0.18
Casein	1.00	1.00	1.00
Mineral mix	0.3	0.3	0.3
Vitamin mix	1.00	1.00	1.00
Choline bitartrate	0.25	0.25	0.25

**Table 2:** Concentrations (*μ*mol/g wet weight; mean ± SD) of acetate, propionate, and butyrate in fecal pellets at week 4.

	AIN	Taro^*∗*^	Flour	Starch
Acetate	4.27 ± 1.20^†‡⸸^	10.15 ± 4.94^†^	12.83 ± 5.73^‡^	7.47 ± 1.29^⸸^
Propionate	1.08 ± 0.26^†‡⸸^	3.33 ± 2.25^†^	4.04 ± 2.87^‡^	2.62 ± 0.96^⸸^
Butyrate	0.19 ± 0.18^†‡⸸^	0.94 ± 0.62^†^	0.69 ± 0.50	1.18 ± 0.63^⸸^

^*∗*^Taro flour and taro starch together. ^†‡⸸^Symbols within rows that are equal indicate significant differences (*p* < 0.01).

## Data Availability

The datasets obtained and/or analyzed during the current study are available from the corresponding author on reasonable request.
